# A selection study on a laboratory-designed population of salmon lice (*Lepeophtheirus salmonis*) using organophosphate and pyrethroid pesticides

**DOI:** 10.1371/journal.pone.0178068

**Published:** 2017-05-22

**Authors:** Elena Myhre Jensen, Sigmund Sevatdal, Marit Jørgensen Bakke, Kiranpreet Kaur, Tor Einar Horsberg

**Affiliations:** 1 Norwegian University of Life Sciences, Faculty of Veterinary Medicine, Sea Lice Research Centre, Oslo, Norway; 2 VESO, Oslo, Norway; Institute of Marine Research, NORWAY

## Abstract

Resistance towards antiparasitic agents in the salmon louse (*Lepeophtheirus salmonis*) is a widespread problem along the Norwegian coast, reducing treatments efficacies and slowing down the envisioned expansion of Norwegian salmon production. The present study was conducted in order to assess the efficacies of two of the most widely used anti-parasitic substances–azamethiphos and deltamethrin–as well as assessing the benefit of having a resistant genotype compared to being fully sensitive when exposed to one of these substances. Atlantic salmon were exposed to a mix of salmon lice copepodids from a fully sensitive, a double resistant and a multi-resistant strain. Once the lice reached pre-adult stages, one group was exposed to 100 μg/L azamethiphos for 60 minutes, the other to 2 μg/L deltamethrin for 30 minutes, and the last was kept in a seawater control. Detached lice were collected at a series of time points following exposure, and all lice (immobilized and surviving) were analysed for both pyrethroid (sensitive “S” and resistant “R”) and azamethiphos (fully sensitive “SS”, heterozygous resistant “RS” and fully resistant “RR”) resistance markers. We found that the efficacies of deltamethrin on parasites with genotype S and R were 70.3 and 13.2%, respectively. The overall efficacy of the deltamethrin treatment was 32.3%. The efficacies of azamethiphos on parasites with genotype SS, RS and RR were 100, 80 and 19.1%, respectively. The overall efficacy of the azamethiphos treatment was 80.4%. Survival analyses revealed that the median survival time in deltamethrin-sensitive and–resistant parasites were 16.8 and >172 hours, respectively. The differences were even more pronounced in the azamethiphos-treated group, where SS, RS and RR parasites survived for 0.26, 6.6 and >172 hours, respectively. The substantial differences in survival between sensitive and resistant lice following treatment demonstrate the ability of medicinal treatments to drive genetic selection towards a much more resistant salmon lice population within a very short time span if there is no influx of sensitive genotypes.

## Introduction

The salmon louse (*Lepeophtheirus salmonis*) has within the last four decades become an extensive problem in Norwegian salmon farming, and the cause of massive expenses every year [1,2]. Salmon lice are naturally occurring marine parasitic copepods that feed on the skin, mucus and blood of their salmonid hosts. Persistent and extensive infestations can lead to stress, anemia, reduced growth, osmoregulatory issues, wounds of the skin of the host—which can act as gateways for secondary infections—as well as interfering with immune responses to such infections [3–6]. Prolonged infections may result in reduced host survival.

Strict regulations are in place to ensure that salmon farms in Norway keep the number of adult female lice below a permitted level per fish; if this level is exceeded action must be taken to lower the infection levels. This level is currently set at 0.5 lice per fish [7]. The Norwegian Food Safety Authority closely monitors production sites, ensuring that weekly counts are completed and lice levels are acceptable. Furthermore, they have the authority to demand a reduction or cessation in production if a location cannot stay below the set infection limit [8]. The most common strategy for controlling salmon lice levels is by medicinal interventions, such as bath treatments with organophosphates or pyrethroids.

Organophosphates were introduced as medicines for use in aquaculture in the early 1980’s, with the first reports of lower treatment efficacies of organophosphates surfaced as early as in 1991 [9]. Pyrethroids, which then became the most used antiparasitic substances in the industry, started declining in efficacy in the late 90’s. Today, reduced efficacies of most available medicines against salmon lice have been reported [10]. Currently, medicinal control methods are essential in aquaculture, although several non-medicinal control methods are emerging [10].

Organophosphates (OP) work by binding to and effectively inactivating enzymes in postsynaptic nerve membranes, the acetylcholinesterases (AChE). AChE’s role is to break down the signaling molecule acetylcholine in order to end nerve signals at the appropriate time. When OPs inactivate the AChE, the signal molecule is not broken down, and the post-synaptic signals are not discontinued, resulting in excitation, paralysis and death of the parasite. Pyrethroids interact with voltage gated sodium channels in the axons of nerve cells in the louse, affecting both their inactivation and activation. The nerve cells are therefore incapable of sending signals normally, and the louse is killed [11,12].

Just as the aforementioned agents work by different mechanisms, resistance has also developed differently. Resistance towards organophosphates has been linked to a *Phe362Tyr* mutation in the AChE coding gene, resulting in the production of an altered protein that is less inhibited by OP, enabling normal nerve signaling in the presence of the substance [13]. This mutation is wide-spread in Norwegian salmon louse populations [14,15]. For pyrethroids, several factors are likely to be involved in resistance, and only parts of the picture have seemingly been uncovered thus far. This is supported by the fact that one study has indicated the involvement of cytochrome P450 in the detoxification of pyrethroids [16], another identified a point mutation in the gene coding for the voltage-gated sodium channel [17], a finding that later could not be reproduced. Whilst yet another study pointed to resistance associated biomarkers in the mitochondrial genome [18].

Resistance caused by changes in the genetic material is heritable and as holders of the resistant genotype will have higher survival rates following exposure to the substance in question, it can lead to genetic selection [9]. Fully sensitive individuals, those lacking the mutations yielding resistance, are more likely to die following exposure to the treatment chemical than the resistant or less sensitive individuals. This in turn shifts the genotype frequencies in the population towards increased resistance. The next generations of salmon lice will then very likely carry the resistance alleles, and fully sensitive individuals become increasingly rare.

In this study, the aim was to observe the efficacies of the two widely utilized antiparasitic agents deltamethrin (pyrethroid) and azamethiphos (organophosphate) on a laboratory created salmon lice population, and assessing the advantage of holding a specific allele type (resistance marker) for survival of a standard treatment with one of these agents.

## Materials and methods

### Salmon lice population

This study was approved by the Norwegian Animal Research Authority, approval number ID4818. The study was conducted at the VESO Vikan research laboratory near Namsos, Norway. A laboratory designed salmon lice population was established by mixing copepodids from three populations that differed in resistance status. One population fully sensitive to pyrethroids, organophosphates and hydrogen peroxide collected in 2010 from Northern Norway; one population resistant to both pyrethroids and organophosphates collected in 2009 from mid-Norway; and one multi-resistant (pyrethroids, organophosphates and hydrogen peroxide) population collected in 2013 from mid-Norway. The populations were held in continuous culture as described by Hamre et al [19]. The sensitivity of the strains were determined by conducting bioassays according to the protocol for pyrethroids described by Sevatdal & Horsberg [20], and adapted to azamethiphos by using 60 minutes exposure. The concentrations immobilizing 50% of the parasites (EC50) were 2.1 μg azamethiphos/L and <0.2 μg deltamethrin/L for the sensitive strain. For the double-resistant strain it was 62 μg azamethiphos/L and 3.3 μg deltamethrin/L; and >100 μg azamethiphos/L and 58 μg deltamethrin/L for the multi-resistant strain. Egg strings collected from female salmon lice of different strains were hatched and allowed to develop to infective copepodids as described by Hamre et al [19] before mixing them in a tentative proportion of 25% sensitive, 50% double-resistant and 25% multi-resistant.

### Infestation

Atlantic salmon postsmolts of similar size (255 individuals, weighing approximately 182 grams) kept in sea water (flow-through, salinity: 33.1 parts per thousands) at 12°C were infected with 3170 copepodids in a 1400 litre tank where the volume was reduced to 700 litres during infestation. Infestation was performed in static seawater with aeration for 60 minutes, after which normal water flow was restored. This gave an approximate average of 5 preadult lice per fish, meaning that approximately 40% of the copepodids developed to the pre-adult stages.

### Treatments

Nine days prior to the selective treatments, three groups of 50 fish each were randomly distributed to separate 200 litre tanks supplied with running seawater that was controlled each day and regulated manually according to fish weight, feed uptake and estimated weight gain. Effluent water was monitored in order to maintain a minimum of 70% oxygen saturation. The treatments were conducted using the products described in Table 1. The test substances were prepared by removing 10 litres of water from the treatment tank and adding either 40 grams of Salmosan™ (20 grams of azamethiphos), or 40 μL Alphamax™ (400 μg deltamethrin). After thorough mixing, the 10 litres containing the treatment was added to the designated treatment tank. Oxygen was supplied through an air-stone in the tank. Treatment was terminated by rapidly draining approximately 2/3 of the tank water, followed by restoration of full water flow. A control group of 50 fish was handled the same way as the treated groups (60 minutes “exposure”), however no treatment was added. The remaining fish were used for studies not addressed here.

**Table 1:** Description of veterinary products and treatments.

Veterinary product	Active substance	Concentration	Treatment method	Recommended dose
AlphaMax (Pharmaq)	Pyrethroid; deltamethrin	1% (w/v)	Bath, single treatment	2 μg/L (deltamethrin) for 30 min
Salmosan (Fish Vet Group)	Organophosphate; azamethiphos	50% (w/w)	Bath, single treatment	100 μg/L (azamethiphos) for 60 min

The selective treatments were performed once a majority of the salmon lice had reached the pre-adult II mobile stages. The recommended treatment concentrations (Table 1) were used for both treatment regimes.

### Sampling

A filter (100 μm mesh size) was fitted to the drain of each tank just before the treatment commenced. Detached salmon lice were collected from the filter and registered at 10, 20, 30, 60, 240 and 1440 minutes (24 hours) following the treatment event. This was accomplished by draining 33 litres of water from the bottom of the tanks. The drained and filtered water was returned to the respective tanks to maintain the required volume. Collected parasites were stored in 70% ethanol until genotyping was possible. After the first 24 hours, the number of detached parasites found in the filter was registered daily. These parasites were not collected for genotyping. The study was terminated 7 days after the selective treatments. All fish were euthanized by an overdose of benzocaine ((Benzoak, ACD), 600 mg benzocaine/L) and all parasites surviving up to that time point were sampled and stored in 70% ethanol for genotyping.

### Genotyping

Salmon lice from the groups treated with azamethiphos and deltamethrin were analysed for genetic markers of resistance using PatoGen’s commercial assay, marketed as LiveAdvisor™ (http://www.patogen.no/vare-tjenester/liceadvisor/) by PatoGen Analyse AS, Ålesund, Norway. This is a standard TaqMan assay for rapid and high-throughput screening of resistance markers. The pyrethroid-marker test targeted a resistance-associated single nucleotide polymorphism (SNP) in the cytochrome b gene in the mitochondrial genome, described in a patent application by Nilsen and Espedal [18]. On this basis, the parasites were classified as either carrying the resistance marker (R) or not (S). The organophosphate-marker test targeted the mutation *Phe362Tyr* in the ace1a gene of *Lepeophtheirus salmonis*, described by Kaur et al. [13]. Based on this assay, each parasite could be classified as homozygote wild type (SS; *Phe362/Phe362*), heterozygote (RS; *362Tyr/Phe362*) or homozygote mutated (RR; *362Tyr/362Tyr*).

In the azamethiphos treated group, 277 of 281 parasites were genotyped (98.6%). In the deltamethrin treated group, 216 of 229 parasites were genotyped (94.3%). Genotyped lice were from both the dead and surviving individuals. The samples that were not genotyped were either samples collected between 24 hours and 7 days after treatment, or samples where the extracted RNA was of poor quality. From the control group, only a small subset of the collected samples (34 of 261 samples, 13%) were genotyped.

### Statistics

Detached lice collected from the filter were classified “immobile” at the given sampling times within the respective treatment groups. The lice still attached to the fish at 7 days post treatment were classified “survivors”. Results were used to calculate treatment efficacies (or background mortality for the control group):

$$Treatment\;efficacy\;(\%)=\frac{total\;number\;of\;immobilized\;lice}{total\;number\;of\;lice}*100$$ (1)

These data were then used for the Kaplan-Meier survival analysis for each of the groups, i.e. the probability of lice surviving in a given time period for each group [21]. Lice remaining on the fish at 7 days post treatment were subjected to right censoring.

Thereafter, data from the two groups treated with either deltamethrin or azamethiphos were sub-grouped into their respective genotypes (R and S in the deltamethrin group, and RR, RS and SS in the azamethiphos group). These data were then used to determine treatment efficacies on the respective genotypes, and separate survival analyses for the genotypes were conducted in the same way as described above.

Pearson’s chi squared tests for independence were used to compare treatment efficacies, first between the control group, the azamethiphos treated group and the deltamethrin treated groups, and then for the different genotypes within the azamethiphos and the deltamethrin groups.

All statistical analyses were conducted in JMP Pro 12.1.0 (SAS Institute).

## Results

### Genotype distribution

The distribution of genotypes in the different groups has been presented in Table 2. For the pyrethroid resistance markers, the R genotype dominated (74.4%); and for the azamethiphos resistance marker, the SS genotype was most prevalent (47.7%), followed by the RS genotype (36.9%). All combinations of genotypes were present (Table 3). The most prevalent combination of genotypes was R-RS (33.3%), followed by R-SS (29.3%), S-SS (18.4%) and R-RR (11.8%). Combinations between the S genotype of the pyrethroid resistance marker and RS or RR genotype of the azamethiphos resistance marker were the rarest, both occurring in only 3.6% of the lice.

**Table 2:** Distribution of genotypes in the 527 parasites genotyped.

No. within group Frequency (%) in population	Pyrethroid resistance genotypes		Organophosphate resistance genotypes		
	S	R	SS	RS	RR
Deltamethrin	64	152	93	86	36
Frequency (%)	29.6	70.4	43.3	40.0	16.7
Azamethiphos	61	216	140	95	42
Frequency (%)	22.0	78.0	50.5	34.3	15.2
Control	10	24	18	13	3
Frequency (%)	29.4	70.6	52.9	38.2	8.8
All	135	392	251	194	81
Frequency (%)	25.6	74.4	47.7	36.9	15.4

**Table 3:** Distribution of combinations of pyrethroid and organophosphate resistance genotypes in the 527 parasites genotyped.

No. within group Frequency (%) in population	Combinations of genotypes					
	S-SS	S-RS	S-RR	R-SS	R-RS	R-RR
Deltamethrin	43	11	10	50	75	26
Frequency (%)	20.0	5.1	4.7	23.3	34.9	12.1
Azamethiphos	46	6	9	94	89	33
Frequency (%)	16.6	2.2	3.2	33.9	32.1	11.9
Control	8	2	0	10	11	3
Frequency (%)	23.5	5.9	0.0	29.4	32.4	8.8
All	97	19	19	154	175	62
Frequency (%)	18.4	3.6	3.6	29.3	33.3	11.8

The first column of Table 2 indicates the treatment groups and all groups combined. The second column gives the number of parasites with the sensitive (S) and resistant (R) pyrethroid resistance genotype within the treatment groups, in the control group and in all groups combined. Below each group the frequency (%) at which each genotype is found in that particular group has been provided. This is repeated for the organophosphate resistance genotypes (SS, RS and RR) in the third column, as all 527 lice were genotyped for both the pyrethroid resistance marker and the organophosphate resistance marker. Note that 1 louse in the deltamethrin treated group was not analysed for organophosphate genotype, only pyrethroid genotype.

Table 3 provides the numbers and frequencies of the combinations of pyrethroid resistance markers (S and R) and organophosphate resistance markers (SS, RS and RR) in the two treatment groups, the control group and for all groups combined.

### Treatment efficacies

The treatment efficacy of deltamethrin was 32.3% in the group as a whole (Table 4), 70.3% on parasites not carrying the resistance-associated SNP (S) and 13.2% on parasites carrying this SNP (R) (Table 5). For azamethiphos, the efficacy was 100% in the homozygous sensitive (SS) individuals, 80.0% in heterozygous resistant (RS) individuals, and 19.1% in the homozygous resistant (RR) individuals (Table 5), or 80.4% in the group as a whole (Table 4).

**Table 4:** Effect of the treatments: Deltamethrin, azamethiphos and control.

Treatment	No. of immobilized	No. of surviving	% efficacy of treatment	Chi-square parameter (Pearson)	p-value
Deltamethrin	74	155	32.3	42.4	<0.001
Azamethiphos	226	55	80.4	279.4	<0.001
Control	23	238	8.8		

The significance level for both chemical treatments was tested against the background mortality in the control group using a chi-square test with 1 degree of freedom.

**Table 5:** Effect of treatment with deltamethrin on the two deltamethrin genotypes R and S, and the effect of treatment with azamethiphos on the three azamethiphos genotypes RR, RS and SS.

Genotype	No. of immobilized	No. of surviving	% efficacy of treatment	Chi-square parameter (Pearson)	p-value
R	20	132	13.2	69.9	<0.001
S	45	19	70.3		
RR	8	34	19.1	139.4	<0.001
RS	76	19	80.0	30.5	<0.001
SS	140	0	100.0		

The significance level for the treatment effect on the genotype R was tested against the treatment effect on the genotype S using a chi-square test with 1 degree of freedom.

The significance level for the treatment effect on the genotypes RS and RR was tested individually against the treatment effect on the genotype SS using a chi-square test with 1 degree of freedom.

Difference in survival between the genotypes (R) and (S) in the deltamethrin treated group was highly significant (Pearson’s chi squared; p<0.001, Table 5). Difference in survival between homozygote resistant (RR), heterozygote resistant (RS) and homozygote sensitive (SS) individuals in the azamethiphos group were also significant when the three groups were tested together. When tested against the efficacy on homozygote sensitive parasites (SS), both the heterozygotes (RS) and the homozygote resistant (RR) parasites were significantly less affected by the treatment (Pearson’s chi squared; p<0.001, Table 5).

### Survival analyses

**Fig 1:**
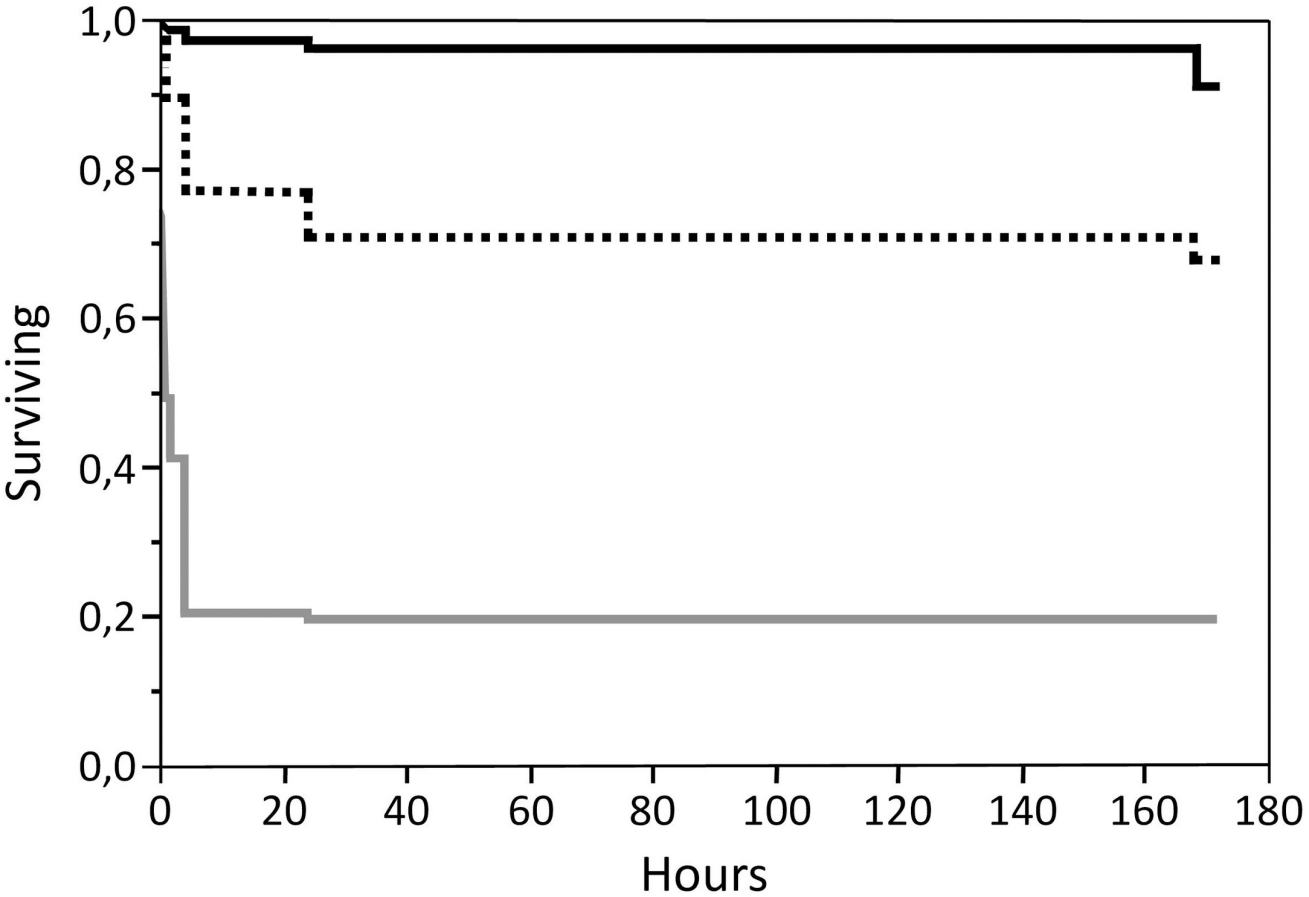
Kaplan-Meier survival plot for groups treated with azamethiphos (grey), deltamethrin (dashed black) and control (black).

**Fig 2:**
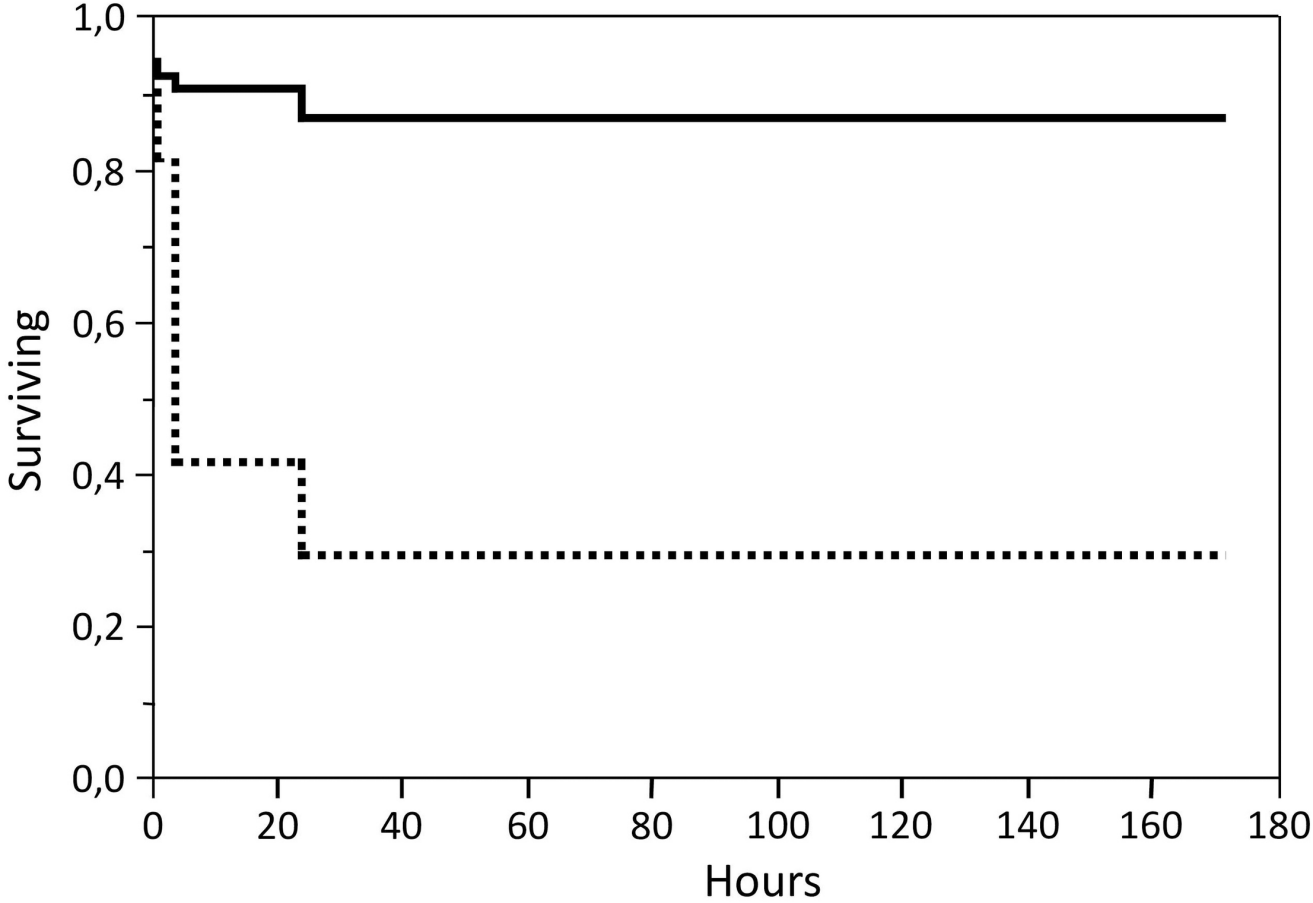
Kaplan-Meier survival plot for the two genotypes R (black) and S (dashed black) in the group treated with deltamethrin.

Survival analyses demonstrated that in the group treated with deltamethrin, the parasites affected by the treatment detached from the fish over the whole 7-days observation period (Fig 1). However, 71.4% had detached within 4 hours after the treatment, and 89.2% within 24 hours. Of the parasites detaching within 4 hours, 73.1% were of the S genotype, and 26.9% of the R genotype (Fig 2). When analysing the full dataset, the calculated time at which the probability of survival was 50% was 16.8 hours for the S genotype, and over 172 hours for the R genotype (Table 6).

**Table 6:** Time (hours) at which the probability of survival is 0.5 for the two different genotypes R (median = >172 h) and S (median = 16.8 h, 95% CI: 8.1 to 35.1) after deltamethrin treatment, and the three different genotypes RR (median = >172 h), RS (median = 6.6 h, 95% CI: 4.1 to 10.7) and SS (median = 0.26 h, 95% CI: 0.23 to 0.29) after azamethiphos treatment.

Treatment	Genotype	Time (h) at which P_(survival)_ = 0.5	95% CI (lower)	95% CI (upper)
Deltamethrin	R	>172	-	-
	S	16.8	8.1	35.1
Azamethiphos	RR	>172	-	-
	RS	6.6	4.1	10.7
	SS	0.26	0.23	0.29

**Fig 3:**
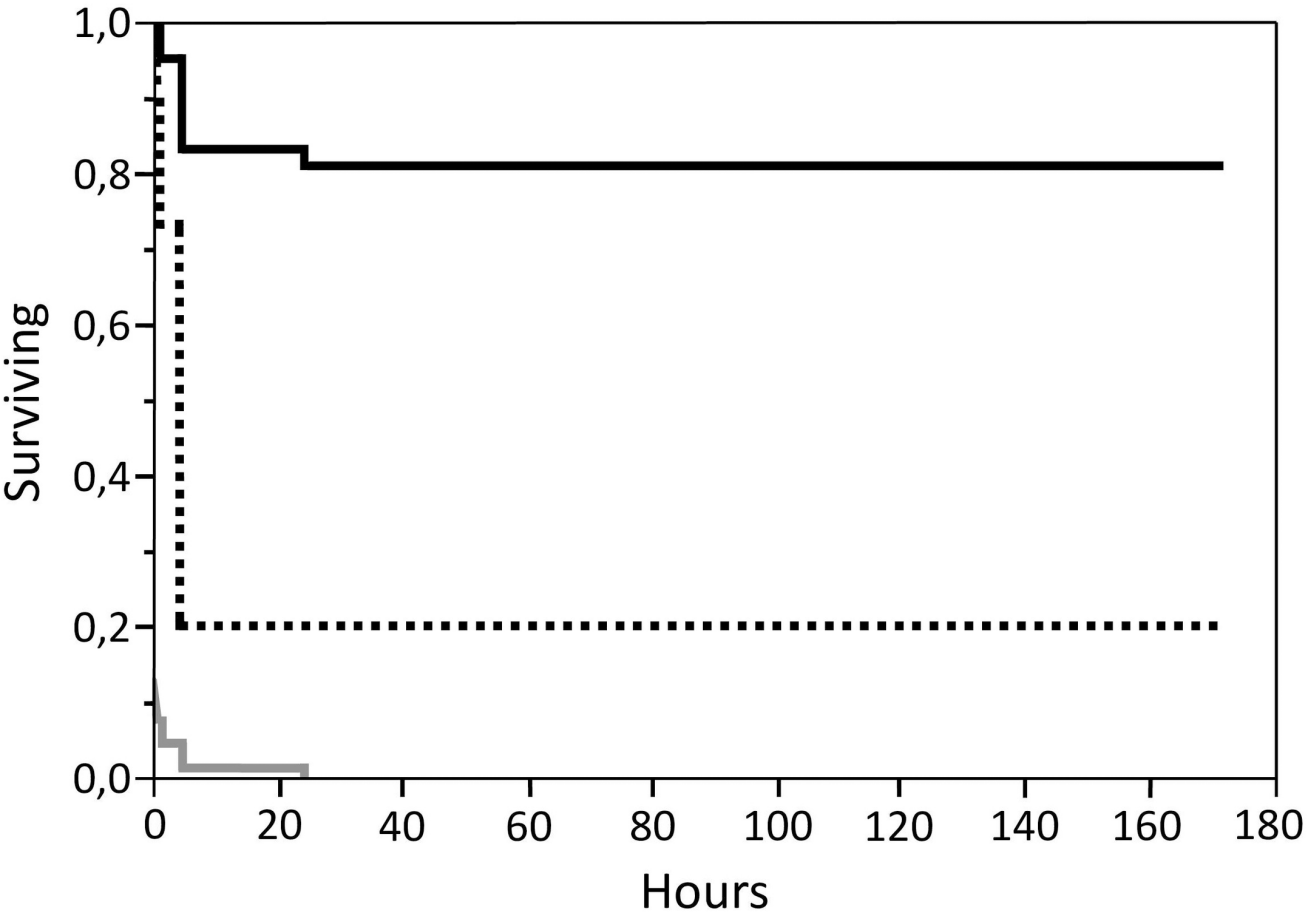
Kaplan-Meier survival plot for the three genotypes RR (black), RS (dashed black) and SS (grey) in the group treated with azamethiphos.

In the azamethiphos treated group, 73.5% of the affected parasites had detached within the first hour, and 99.1% by 4 hours. No detached parasites were seen later than 24 hours after treatment (Fig 1). All 83 parasites that died during the first 10 minutes of treatment were of the SS genotype. Of the parasites detaching within 1 hour, 82.9% were of the SS genotype, 15.9% were RS and 1.2% of the RR genotype (Fig 3). When analysing the full dataset, the calculated time at which the probability of survival was 50% was 0.26 hours (16 min.) for the SS genotype, 6.6 hours for RS and over 172 hours for the RR genotype (Table 6).

### Development in genotype frequencies

The Hardy-Weinberg principle is useful to demonstrate the strength of the genetic drift following experimental treatments with azamethiphos and deltamethrin [22]. As an example, the azamethiphos treatment was 100% efficacious on fully azamethiphos-sensitive parasites (SS), 80% on heterozygous parasites (RS), but only 19% on fully resistant parasites (RR). Consequently, the next generation of parasites will have a different composition of genotypes compared to the parent generation. The Hardy-Weinberg equation is as follows:

$$p^{2}+2pq+q^{2}=1$$ (2)

where *p* equals the frequency of S-alleles and *q* equals the frequency of R-alleles.

Entering the frequencies of allele, calculated from the number of surviving parasites of the different genotypes in Table 5 (S(*p*) = 0.179, R(*q*) = 0.821) into Eq (2), it can be estimated that the frequency of genotypes between the F0 and F1 generation will shift from 50.5 to 3.2% (SS, *p*^*2*^), 34.3 to 29.4% (RS, *2pq*) and 15.2 to 67.4% (RR, *q*^*2*^), assuming random mating and no influx of external parasites. Consequently, the predicted total treatment efficacy of azamethiphos on the F1 generation can be calculated as the sum of efficacy on the individual genotypes:

$$100\;\%\;\mathrm{on}\;\mathrm{SS}\;\left(3.2\;\%\;\mathrm{of}\;F1\;\mathrm{population}\right)+80\;\%\;\mathrm{on}\;\mathrm{RS}\;\left(29.4\;\%\;\mathrm{of}\;F1\;\mathrm{population}\right)+19.1\;\%\;\mathrm{on}\;\mathrm{RR}\;\left(67.4\;\%\;\mathrm{of}\;F1\;\mathrm{population}\right)=\underline{39.6}\;\%$$

which is a significant decrease from the efficacy on the F0 generation (80.4% efficacy).

The Hardy-Weinberg principle is based on the random merging of two haploid gametes, or Mendelian inheritance. As the pyrethroid-resistance marker is found in a mitochondrial gene it follows maternal inheritance, which implies direct inheritance from mother to offspring. Thus, the Hardy-Weinberg principle could not be applied to these numbers. However, using the numbers from Table 4 and assuming equal proportions of males and females in the population, the predicted efficacy in the F1 generation can be calculated by adding the efficacies on the individual genotypes:

$$70.3\;\%\;\mathrm{on}\;S\;\left(12.6\;\%\;\mathrm{of}\;F1\;\mathrm{population}\right)+13.2\;\%\;\mathrm{on}\;R\;\left(87.4\;\%\;\mathrm{of}\;F1\;\mathrm{population}\right)=\underline{20.4}\;\;\%$$

which is also a significant decrease from the efficacy on the F0 generation (32.3% efficacy).

## Discussion

Since 2008, a major issue in Norwegian salmon farming has been the increasing prevalence of resistance in salmon lice towards the primary antiparasitic substances available in the country [10]. Consequently, staying below the maximum threshold of infection of 0.5 adult female lice per fish has become labor-intensive and very expensive. The Norwegian government is reluctant to let production companies increase their production, and may in fact require heavily affected farms to decrease their production until they are able to effectively control infection levels [8].

The specific treatment efficacy of any substance will naturally depend on the frequency of resistant genotypes in that population prior to treatment. The issue of concern is that resistance towards several substances (multi-resistance) has emerged in some areas along the Norwegian coast [23]. This effectively limits the industry’s ability to alternate between substances if they experience low treatment efficacies from one of them. The level at which resistance in salmon lice can be tolerated will depend on many factors [9], but widespread resistance can undoubtedly restrain the production of the salmon farming industry to a large extent, as well as having many other damaging consequences. One such repercussion is the cross infection that might take place between the farmed salmon and wild salmon. Numbers of wild salmon have declined to roughly half of what they were in the 1980’s, and the farming of salmon has been identified as one of the main culprits [24]. If the resistance issues continue to increase and efficacies of treatments decrease, the lice pressure on wild salmon that either migrate in proximity to farms or encounter runaway farmed salmon will be high [24].

To assess the resistance status towards azamethiphos in this study, a well-described genomic marker was used. The resistance marker towards azamethiphos is strongly associated with azamethiphos resistance, as described by Kaur et al. [13,15] and is likely directly linked to the resistance mechanism. To assess the resistance status towards pyrethroids lice were analysed for a SNP in the mitochondrial gene coding for cytochrome B, according to the patent application of Nilsen and Espedal [18]. While it is unclear whether this SNP is directly involved in the resistance mechanism, the observation that 29.7% of the parasites that survived the deltamethrin treatment were of the S genotype provides weight to the suggestion that it may only be a biomarker associated with the direct mechanism.

A higher overall treatment efficacy was observed in the group treated with azamethiphos (80.4%) compared to the group treated with deltamethrin (32.3%). There was a clear connection between possessing one or more copies of the resistance markers and surviving the selective treatments. This held true for both treated groups (Table 5).

A strong genetic drift towards resistant genotypes could be demonstrated after the experimental treatments with deltamethrin or azamethiphos. This was exhibited through use of the Hardy-Weinberg principle in which the RR genotype was expected to increase in prevalence by 52.2%. Combined with a reduction of the SS genotype by 47.3%, it becomes clear that medicinal treatments in a population that has emerging resistance issues can (and will) act as bottlenecks that make fully sensitive salmon lice decline in numbers.

We observed that the lice that were immobilized by treatment with azamethiphos were very rapidly affected by the substance. Most deaths were registered within the first hour following exposure, and none were observed after 24 hours. The fully sensitive parasites died very rapidly, with a median survival time of only 16 minutes, whereas the heterozygote parasites had a median survival time of 6.6 hours. The efficacy and survival time for the SS genotype coincided with the results of Kaur et al. [13], but the efficacy of the treatment on the RS genotype was higher and the survival time longer than reported in Kaur et al. [13]. The most likely explanation is that in the current study, the treatment time was 60 minutes, while in the study by Kaur et al. [13] it was 30 minutes. This led to a higher efficacy on the RS genotype (80% in the current study versus 40% in the study by Kaur et al. [13]). A small effect on the RR genotype (19.1%) could be seen in the current study, whereas no effect on this genotype was seen in the study by Kaur et al [13].

For deltamethrin, the effect was slower, and deaths were registered across the 7 day study period. The median survival time for the S genotype was 16.8 hours, substantially longer than for both the azamethiphos SS and RS genotypes. This corresponds with anecdotal observations from the field where the efficacy of azamethiphos treatments are claimed to be accurately recorded the day after treatment, whereas no accurate treatment effect following pyrethroid treatments can be done before one week post-treatment.

The resistance markers against both treatments were analysed in all individuals, making it possible to look for correlation between markers for pyrethroid resistance and organophosphate resistance. Survival frequencies in the azamethiphos treated group correlated well with the genotypes (SS, RS and RR), with no effect from the pyrethroid genotypes (S and R). However, for the sample population treated with deltamethrin, 9% of the individuals that were labelled as sensitive (S) based on genotyping, survived the treatment. This result strongly indicates a lack of complete genotype-phenotype correlation for the pyrethroid biomarker used in the present study and other yet unidentified marker(s) might be better associated with resistance towards this group of chemicals. An interesting observation was that all of the surviving S samples were genotyped as RR (n = 10) or RS (n = 9) for azamethiphos, signifying an association of the azamethiphos resistant biomarker to the pyrethroid resistance. Unfortunately, the sample size was too small to draw any definite conclusions. Besides, the sample population was a mix of three populations, wherein two populations were resistant towards both deltamethrin and azamethiphos. Thus, co-selection of the azamethiphos resistance biomarker with the unidentified pyrethroid marker(s) could be a plausible explanation for the current observation. Only a few published studies have looked at a possible link between organophosphate and pyrethroid resistance in arthropods. The acetylcholine esterase (AChE) activity following deltamethrin treatment has been shown to be reduced in both *Daphnia magna* [25] and the black tiger shrimp *Penaeus monodon* [26], whereas the AChE activity was increased in honeybees surviving a deltamethrin treatment [27]. In addition, the pyrethroids can affect the release of acetylcholine in the rat brain [28]. These observations may lend some support to a possible cross-resistance between azamethiphos and pyrethroids. However, further studies are warranted to validate this possibility of co-selection and furthermore a possible cross-resistance between azamethiphos and deltamethrin in the salmon lice.

As a total of 7 lice in the deltamethrin treated group were found detached during the 24–144 hour period following treatment, but were not genotyped, a validation was conducted to be certain that these lice would not significantly affect the results. As their genotypes were unknown, 7 lice were first added to the number of detached lice in the resistant group (R), followed by a new run of contingency analysis. The same was done in the sensitive (S) group in a separate analysis. The results from these analyses were compared to the original analyses, and it was concluded that these results did not significantly alter the outcome (S1 File).

Pens in a salmon farm are not closed structures, but semipermeable. This implies that salmon lice from neighboring pens, neighboring farms or even from greater distances, carried by water currents, can be introduced into the population in a single pen [29]. Thus, a population of salmon lice will rarely be isolated. Gene flow will in most cases be upheld, which also includes the introduction of new sensitive individuals. However, the observed increase in tolerant salmon lice along most of the Norwegian coast suggests that the selection pressure constituted by medicinal treatments is stronger than the gene flow of sensitive alleles into those areas.

As long as some individuals with the heterozygous genotype survive, the sensitive genotype will not be eradicated. Conversely, if the sensitive genotype is eradicated, resistance would be impossible to reverse. If the resistance allele comes with a high fitness cost, it is not likely to be selected for when the lice are not exposed to the substance in question, and selection would instead be directed towards the sensitive genotype (which would increase in numbers). However, if there is a small fitness cost associated, the mutant alleles can prevail in the population in the absence of treatment events. The majority of reported mutations in AChE are point mutations that only slightly alter the protein, and in turn results in a low level of associated fitness cost, which could be the driving force behind their existence in the population without any selection pressure [30]. This hypothesis has been supported in the study by Kaur et al. [13], where the authors found low frequencies (4%) of salmon lice heterozygous for mutation *Phe362Tyr* in a population that had not been exposed to OPs in eight years in the field, and a further fifteen generations in cultivation. Fallang et al. [31] hypothesize that the presence of resistance towards one substance is likely to have little effect on fitness, but that multi-resistance, that is salmon lice with resistance towards two or several substance groups, can lead to a stronger negative effect on fitness for the holder. Conducting further studies into fitness costs connected to resistance towards the medicinal substances in use today, as well as multi-resistant salmon lice would be appropriate.

Computer modelling is a growing field in sea lice research. Models are being designed to evaluate management strategies [32], to observe the effects of natural refugia (wild salmon populations) on resistance development on a salmon farm [33], and to use locality and number of treatments in that area to predict the efficacy of antiparasitic agents against adult lice [14], to mention a few applications. A well-designed prediction model may become an invaluable tool for a production site, as it enables evidence-based decisions to be made regarding lice control strategies, managing, economical efficiency and to increase the welfare of the farmed fish. The results from the current study could provide valuable input parameters in such a model.

## Conclusions

The specific treatment efficacy of any substance will naturally depend on the frequency of resistant genotypes in that population prior to treatment. In this study, neither azamethiphos nor deltamethrin were able to remove all attached salmon lice from the hosts at the recommended doses. Further, we demonstrated that there are significant differences in survival between genotypes following a single treatment with deltamethrin or azamethiphos, thus treatments are able to alter the genotype frequencies in a salmon lice population within relatively short time frames. The surviving part of the population will have a high occurrence of resistant genotypes, demonstrating that such medicinal treatments can act as strong driving forces towards resistance development. Relying too heavily on one or only a few substances against salmon lice can thus act as a catalyst for selection towards highly resistant populations [9].

## Supporting information

S1 File Supporting information file including information on all analysed lice, all registered detachment time points and genotyping data, as well as the statistical analyses conducted. (XLSX)
