# Correction: A selection study on a laboratory-designed population of salmon lice (*Lepeophtheirus salmonis*) using organophosphate and pyrethroid pesticides

**DOI:** 10.1371/journal.pone.0181388

**Published:** 2017-07-10

**Authors:** The PLOS ONE Staff

## Notice of Republication

This article was republished on May 26, 2017, as the original article title was published in error. The correct title, “A selection study on a laboratory-designed population of salmon lice (*Lepeophtheirus salmonis*) using organophosphate and pyrethroid pesticide,” was incorrectly published as “A selection study on a laboratory-designed population of salmon lic (*Lepeophtheirus salmonis*) using organophosphate and pyrethroid pesticides.” The publisher apologizes for the error.
